# ANALYZING LONGEVITY TRENDS: AGE-SPECIFIC CONTRIBUTIONS TO LIFE EXPECTANCY GAINS ACROSS EUROPE

**DOI:** 10.1093/geroni/igae098.0191

**Published:** 2024-12-31

**Authors:** Yeonjung Lee, Iona Tsui, Yongjie Yon

**Affiliations:** Chung-Ang University, Seoul, Republic of Korea; World Health Organization Regional Office for Europe, Copenhagen, Hovedstaden, Denmark; World Health Organization Regional Office for Europe, Copenhagen, Hovedstaden, Denmark

## Abstract

The increase in life expectancy at birth has been apparent as mortality has decreased over the past decades. However, as people live longer, it is important to know which age groups have seen the greatest gains in life expectancy. The objective of this study is to examine how different age groups contribute to longer life expectancy. We applied Arriaga’s (1984) life expectancy decomposition method to life tables for the total population of 50 out of the 53 Member States of the WHO European Region comparing two different time periods. Average life expectancy gains between 1999 and 2019 are 5.08 years, compared to 3.50 years between 1980 and 2000. Decomposing the differences in life expectancy showed that the gains in total life expectancy was increasingly driven by reduced mortality at older ages in many countries, especially those with the highest current life-expectancy, though there are variation among the countries. There are also differences between men and women. The differences in life expectancy gains between men and women range from 3.05 to 10.43 years and there are the largest differences in Eastern European countries. There is still room for improvement in health beyond age 70, as older age groups are contributing more to increasing longevity. The contribution of increased longevity after age 70 to total improvement in life expectancy at birth is above 30% for more than half of the countries, and this measure is expected to rise as population ageing continues to accelerate.

